# Computational Approach to Identifying Universal Macrophage Biomarkers

**DOI:** 10.3389/fphys.2020.00275

**Published:** 2020-04-08

**Authors:** Dharanidhar Dang, Sahar Taheri, Soumita Das, Pradipta Ghosh, Lawrence S. Prince, Debashis Sahoo

**Affiliations:** ^1^Department of Computer Science and Engineering, University of California, San Diego, San Diego, CA, United States; ^2^Department of Pediatrics, University of California, San Diego, San Diego, CA, United States; ^3^Department of Pathology, University of California, San Diego, San Diego, CA, United States; ^4^Departments of Medicine and Cellular and Molecular Medicine, University of California, San Diego, San Diego, CA, United States; ^5^Moores Cancer Center, San Diego, CA, United States; ^6^Rady Children’s Hospital, San Diego, CA, United States

**Keywords:** macrophage, CAD, gene expression, biomarker, Boolean analysis

## Abstract

Macrophages engulf and digest microbes, cellular debris, and various disease-associated cells throughout the body. Understanding the dynamics of macrophage gene expression is crucial for studying human diseases. As both bulk RNAseq and single cell RNAseq datasets become more numerous and complex, identifying a universal and reliable marker of macrophage cell becomes paramount. Traditional approaches have relied upon tissue specific expression patterns. To identify universal biomarkers of macrophage, we used a previously published computational approach called BECC (Boolean Equivalent Correlated Clusters) that was originally used to identify conserved cell cycle genes. We performed BECC analysis using the known macrophage marker CD14 as a seed gene. The main idea behind BECC is that it uses massive database of public gene expression dataset to establish robust co-expression patterns identified using a combination of correlation, linear regression and Boolean equivalences. Our analysis identified and validated FCER1G and TYROBP as novel universal biomarkers for macrophages in human and mouse tissues.

## Introduction

Macrophages are specialized cells involved in the detection, phagocytosis and destruction of bacteria and other harmful organisms. In addition, they can also present antigens to T cells and initiate inflammation by releasing molecules (known as cytokines) that activate other immune effector cells. Further, Macrophages migrate to and circulate within almost every tissue, patrolling for pathogens or eliminating dead or damaged cells. Critical for immune protection and tissue homeostasis, macrophage functions can be corrupted in multiple disease processes (Wynn et al., 2013). Disruption of normal macrophage biology is a hallmark of many diseases, including diabetes (Huang et al., 2010; Eguchi et al., 2012), asthma (Gordon, 2003), metastatic cancer (Qian and Pollard, 2010), tissue fibrosis (Murray and Wynn, 2011), and chronic inflammation (Kamada et al., 2008; Hansson and Hermansson, 2011; Murray and Wynn, 2011). These characteristics make understanding macrophage biology vital for studying disease pathogenesis. Macrophages function in both tissue repair during homeostasis and in the innate immune response (Wynn et al., 2013). Inflammation, which can be triggered by infection, is accompanied by a massive expansion of macrophages in affected tissues. Macrophages resulting from inflammation were thought to derive from hematopoietic stem cells in the bone marrow. However, a recent study shows that macrophages can initiate cell division and can self-replicate within various tissues (Hoeffel and Ginhoux, 2015; Dick et al., 2019). These functions are essential to protect against microbial infection and to maintain tissue homeostasis (Sieweke and Allen, 2013). These critical functionalities have propelled researchers to better understand macrophage biology.

Recent advances in high-throughput sequencing technologies have facilitated large collections of biological datasets. These large datasets have enabled efforts to model the complexities of macrophage biology. Macrophage expression data contains diverse and variable patterns, even when examining established and traditional markers of macrophage identity. Difficulty and variability in experimental techniques and complex purification strategies may have limited the ability to identify a reliable universal macrophage biomarker. Commonly used markers for macrophages such as CD14 (Ziegler-Heitbrock and Ulevitch, 1993), ITGAM (Swirski et al., 2009), CD68 (Falini et al., 1993), and EMR1 (Austyn and Gordon, 1981) have shown variable expression patterns in different tissues.

Large scale genomic profiling studies have identified differences in macrophage gene expression based on developmental stage, tissue location, and disease process. Novel informatic analysis of these large datasets could leverage the diversity of gene expression data and identify specific patterns and pathways regulating macrophage biology. Collombet et al. (2017) have proposed a dynamic logical model of blood cell macrophages using a limited number of gene expression datasets. Such a model may not be generalized as the authors did not consider a wide range of datasets. Boolean modeling has been proposed to study the complexities of macrophage polarization and activation in experimental disease models and *in vivo* systems by incorporating large numbers of available datasets (Rex et al., 2016; Palma et al., 2018). Boolean modeling of the NFκB pathway in bacterial lung infection has been explored (Cantone et al., 2017).

In this paper, we will discuss a new strategy that leverages massive amounts of public gene expression dataset to capture robust co-expression patterns. Our strategy uses traditional correlation and linear regression and augment the results by new Boolean approaches which reliably distinguish asymmetric vs. symmetric relationships. Asymmetric relationships are discarded, and symmetric relationships are used to identify genes that perfectly mirror each other with respect to their gene expression pattern.

## Materials and Methods

### Data Collection and Annotation

Publicly available microarray databases in Human U133 Plus 2.0 (*n* = 25,955, GSE119087), Mouse 430 2.0 (*n* = 11,758, GSE119085) Affymetrix platform were downloaded from National Center for Biotechnology Information (NCBI) Gene Expression Omnibus (GEO) website (Edgar et al., 2002; Barrett et al., 2005, 2013). Gene expression summarization was performed by normalizing each Affymetrix platform by RMA (Robust Multichip Average) (Irizarry et al., 2003a, b). One hundred ninety-seven published macrophage samples from seven series assayed on the Human U133 Plus 2.0 (GPL570), Human U133A 2.0 (GPL571) and Human U133A (GPL96) platforms were re-analyzed and deposited in GEO with accession no GSE134312. RMA was used to normalize the macrophage CEL files using a modified CDF file that contains the shared probes among the three different platforms. The global human dataset GSE119087 included 106 macrophage samples from GSE134312 dataset. Mouse dataset GSE119085 was also annotated with 327 macrophage samples that were deposited in GEO with accession no GSE135324. In addition to the above training datasets, several human and mouse validation datasets were assembled from GEO. We validate our markers in 39 distinct highly purified mouse hematopoietic stem, progenitor, and differentiated cell populations covering almost the entire hematopoietic system: Gene Expression Commons (GEXC, GSE34723, *n* = 101) (Seita et al., 2012). In addition to GEXC, we also used ImmGen datasets that are also purified mouse blood cells (GSE15907 and GSE127267) (Painter et al., 2011; Yoshida et al., 2019).

We put together four purified human macrophage datasets: (GSE35449, *n* = 21) (Beyer et al., 2012), (GSE85333, *n* = 185) (Regan et al., 2018), (GSE46903, *n* = 384) (Xue et al., 2014), (GSE55536, *n* = 33) (Zhang et al., 2015).

GSE35449 (PBMC): CD14 + monocytes were isolated from Peripheral blood mononuclear cells (PBMC) using CD14-specific MACS beads and cultured in 6-well plates in media and provided various stimuli: IFN-γ, TNF-α, ultrapure LPS, IL-4, IL-13, or combinations thereof.

GSE85333 (PBMC): Primary human CD14+ monocytes were isolated from the whole blood of six donors (three males, three females). These were transformed in macrophages through CSF-1 stimulation over a week. Cells were then subject to an inflammatory stimulus with LPS or IFNa and without any inflammatory stimulus.

GSE46903 (PBMC): Human monocytes were purified from peripheral blood mononuclear cells by MACS, followed by stimulation with GM-CSF or M-CSF for 72 h.

GSE55536 (iPSDMs and PBMC): Transcriptome analyses of human induced pluripotent stem cell-derived macrophages (IPSDMs) and their isogenic human peripheral blood mononuclear cell-derived macrophage (HMDM) counterparts.

To validate our results in the mouse, we put together four diverse mouse macrophage datasets: (GSE82158, *n* = 163) (Misharin et al., 2017), (GSE38705, *n* = 511) (Orozco et al., 2012), (GSE62420, *n* = 56) (Grabert et al., 2016), and (GSE86397, *n* = 12) (Han et al., 2017).

GSE82158 (interstitial and alveolar): Monocytes, interstitial macrophages, and alveolar macrophages were isolated from naïve mice and RIPK3^–/–^ mice.

GSE38705 (intraperitoneal lavage): Primary macrophages were harvested using four mice per strain which were exposed to either LPS or OxPAPC.

GSE62420 (Brain Microglia): Microglia cells were extracted from 4 regions: cerebellum, cortex, hippocampus, striatum using a magnetic bead-based approach.

GSE86397 (Liver Kupffer cells): Primary Kupffer cells isolated from mouse liver were treated with lipopolysaccharides or IL-4 and the gene expression patterns were analyzed by microarray.

We validated our results on following tissue resident macrophages in human: tumor associated macrophage (GSE117970, *n* = 116) (Cassetta et al., 2019); lung alveolar macrophages (GSE116560, *n* = 68) (Morrell et al., 2019); lung alveolar macrophages (GSE40885, *n* = 14) (Reynier et al., 2012); cardiac macrophages (GSE119515, *n* = 18) (Dick et al., 2019); vaginal mucosa and skin macrophages (GSE54480, *n* = 87) (Duluc et al., 2014); skin macrophages (GSE74316, *n* = 77) (Carpentier et al., 2016); peritoneal macrophages (GSE79833, *n* = 12) (Irvine et al., 2016); microglia (GSE1432, *n* = 24) (Rock et al., 2005); adipose tissue macrophages (GSE37660, *n* = 4) (Eto et al., 2013).

To validate our results on single cell RNASeq data we used following datasets: mouse inflammatory airway macrophages (GSE120000, *n* = 1,142) (Mould et al., 2019), mouse CX3CR1-derived macrophage from atherosclerotic aorta (GSE123587, *n* = 5,355) (Lin et al., 2019), mouse dissociated whole lung tissue (GSE111664, *n* = 41,898) (Aran et al., 2019), and renal resident macrophages across species (GSE128993; human *n* = 2,868, mouse *n* = 3,013, rat *n* = 3,935, pig *n* = 4,671) (Zimmerman et al., 2019).

We also examined expression patterns in skin Langerhans cell (GSE49475, *n* = 39) (Polak et al., 2014) and dermal dendritic cells (GSE74316, human *n* = 77, mouse *n* = 74) (Carpentier et al., 2016).

### StepMiner Analysis

StepMiner is a computational tool that identifies step-wise transitions in a time-series data (Sahoo et al., 2007). StepMiner performs an adaptive regression scheme to identify the best possible step up or down based on sum-of-square errors. The steps are placed between time points at the sharpest change between low expression and high expression levels, which gives insight into the timing of the gene expression-switching event. To fit a step function, the algorithm evaluates all possible step positions, and for each position, it computes the average of the values on both side of the step for the constant segments. An adaptive regression scheme is used that chooses the step positions that minimize the square error with the fitted data. Finally, a regression test statistic is computed as follows:

$$F\,s\,t\,a\,t=\frac{\sum_{i=1}^{n}{\left(\hat{X_{i}}-\bar{X}\right)}^{2}/\left(m-1\right)}{\sum_{i=1}^{n}{\left(X_{i}-\hat{X_{i}}\right)}^{2}/\left(n-m\right)}$$

Where *X*_*i*_ for *i=1* to *n* are the values, $\hat{X_{i}}$ for *i=1* to *n* are fitted values. m is the degrees of freedom used for the adaptive regression analysis. $\bar{X}$ is average of all the values: $\hat{X}=\frac{1}{n}*\sum_{j=1}^{n}X_{j}$. For a step position at k, the fitted values $\hat{X_{i}}$ are computed by using $\frac{1}{k}*\sum_{j=1}^{n}X_{j}$ for *i=1* to *k* and $\frac{1}{\left(n-k\right)}*\sum_{j=k+1}^{n}X_{j}$ for *i* = *k* + 1 to *n*.

### Boolean Analysis

**FIGURE 1:**
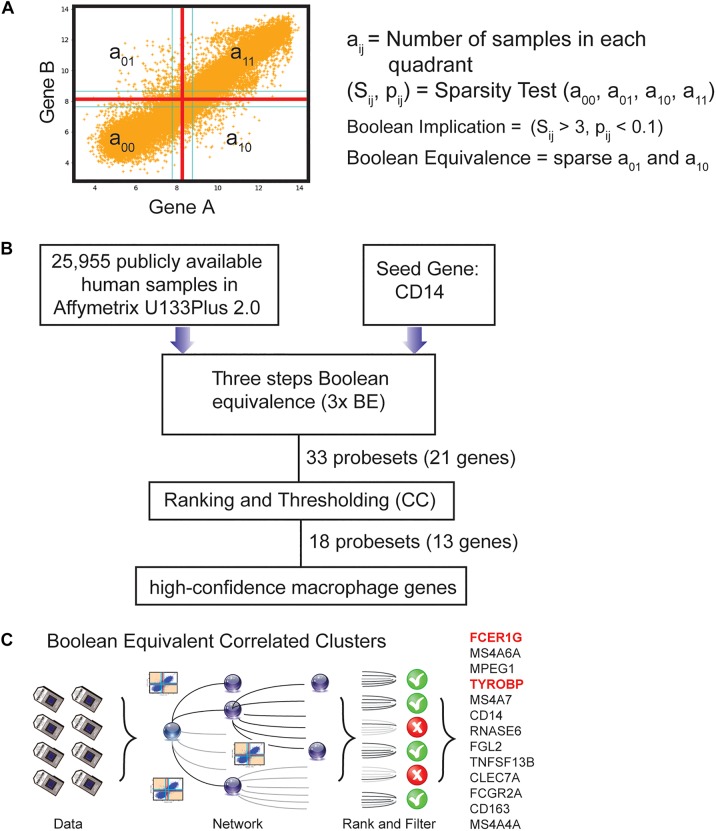
Computational approach to identifying candidate universal macrophage biomarker. **(A)** BooleanNet Statistical test to identify Boolean Implication relationship between gene A and B. Boolean equivalent relationship is found when both a_01_ and a_10_ is sparse. **(B)** A flow chart of the different steps of BECC (Boolean Equivalence Correlated Clusters) to identify robust macrophage biomarker. **(C)** Overview of BECC illustrating input data, building networks, ranking and filtering that finally selected 13 genes.

**Boolean logic** is a simple mathematic relationship of two values, i.e., high/low, 1/0, or positive/negative. The Boolean analysis of gene expression data requires conversion of expression levels into two possible values. The ***StepMiner*** algorithm is reused to perform Boolean analysis of gene expression data (Sahoo et al., 2008). The **Boolean analysis** is a statistical approach which creates binary logical inferences that explain the relationships between phenomena. Boolean analysis is performed to determine the relationship between the expression levels of pairs of genes. The ***StepMiner*** algorithm is applied to gene expression levels to convert them into Boolean values (high and low). In this algorithm, first the expression values are sorted from low to high and a rising step function is fitted to the series to identify the threshold. Middle of the step is used as the StepMiner threshold. This threshold is used to convert gene expression values into Boolean values. A noise margin of twofold change is applied around the threshold to determine intermediate values, and these values are ignored during Boolean analysis. In a scatter plot, there are four possible quadrants based on Boolean values: (low, low), (low, high), (high, low), (high, high). A Boolean implication relationship is observed if any one of the four possible quadrants or two diagonally opposite quadrants are sparsely populated. Based on this rule, there are six different kinds of Boolean implication relationships. Two of them are symmetric: equivalent (corresponding to the highly positively correlated genes), opposite (corresponding to the highly negatively correlated genes). Four of the Boolean relationships are asymmetric and each corresponds to one sparse quadrant: (low ⇒ low), (high ⇒ low), (low ⇒ high), (high ⇒ high). BooleanNet statistics (Figure 1A, Equations listed below; Supplementary Figures S1A,B) is used to assess the sparsity of a quadrant and the significance of the Boolean implication relationships (Sahoo et al., 2008, 2010). Given a pair of genes A and B, four quadrants are identified by using the StepMiner thresholds on A and B by ignoring the Intermediate values defined by the noise margin of 2 fold change (±0.5 around StepMiner threshold). Number of samples in each quadrant are defined as a_00_, a_01_, a_10_, and a_11_ (Figure 1A) which is different from X in the previous equation of F stat. Total number of samples where gene expression values for A and B are low is computed using following equations.

$$n\,A_{l\,o\,w}=\left(a_{00}+a_{01}\right),n\,B_{l\,o\,w}=\left(a_{00}+a_{10}\right),$$

Total number of samples considered is computed using following equation.

$$t\,o\,t\,a\,l=a_{00}+a_{01}+a_{10}+a_{11}$$

Expected number of samples in each quadrant is computed by assuming independence between A and B. For example, expected number of samples in the bottom left quadrant e_00_ = $\hat{n}$ is computed as probability of A low [(a_00_ + a_01_)/total] multiplied by probability of B low [(a_00_ + a_10_)/total] multiplied by total number of samples. Following equation is used to compute the expected number of samples.

$$n=a_{i\,j},\hat{n}=\left(n\,A_{l\,o\,w}/t\,o\,t\,a\,l*n\,B_{l\,o\,w}/t\,o\,t\,a\,l\right)*t\,o\,t\,a\,l$$

To check whether a quadrant is sparse, a statistical test for (e_00_ > a_00_) or ($\hat{n}>n)$ is performed by computing S_00_ and p_00_ using following equations. A quadrant is considered sparse if S_00_ is high ($\hat{n}>n)$ and p_00_ is small.

$$S_{i\,j}=\frac{\hat{n}-n}{\sqrt{\hat{n}}}$$

$$p_{00}=\frac{1}{2}\,\left(\frac{a_{00}}{\left(a_{00}+a_{01}\right)}+\frac{a_{00}}{\left(a_{00}+a_{10}\right)}\right)$$

We used a threshold of S_00_ > 3 and p_00_ < 0.1 to check sparse quadrant. A Boolean implication relationship is identified when a sparse quadrant is discovered using following equation.

BooleanImplication=(S>i⁢j3,p<i⁢j0.1)

A relationship is called Boolean equivalent if top-left and bottom-right quadrants are sparse.

$$Equivalent=\left(S_{01}>3,P_{01}<0.1,S_{10}>3,P_{10}<0.1\right)$$

Boolean opposite relationships have sparse top-right (a_11_) and bottom-left (a_00_) quadrants.

$$Opposite=\left(S_{00}>3,P_{00}<0.1,S_{11}>3,P_{11}<0.1\right)$$

Boolean equivalent and opposite are symmetric relationship because the relationship from A to B is same as from B to A. Asymmetric relationship forms when there is only one quadrant sparse (A low ⇒ B low: top-left; A low ⇒ B high: bottom-left; A high ⇒ B high: bottom-right; A high ⇒ B low: top-right). These relationships are asymmetric because the relationship from A to B is different from B to A. For example, A low ⇒ B low and B low ⇒ A low are two different relationships.

A low ⇒ B high is discovered if bottom-left (a_00_) quadrant is sparse and this relationship satisfies following conditions.

$$Alow\Rightarrow Bhigh=\left(S_{00}>3,P_{00}<0.1\right)$$

Similarly, A low ⇒ B low is identified if top-left (a_01_) quadrant is sparse.

$$Alow\Rightarrow Blow=\left(S_{01}>3,P_{01}<0.1\right)$$

A high ⇒ B high Boolean implication is established if bottom-right (a_10_) quadrant is sparse as described below.

$$Ahigh\Rightarrow Bhigh=\left(S_{10}>3,P_{10}<0.1\right)$$

Boolean implication A high ⇒ B low is found if top-right (a_11_) quadrant is sparse using following equation.

$$Ahigh\Rightarrow Blow=\left(S_{11}>3,P_{11}<0.1\right)$$

For each quadrant a statistic S_*ij*_ and an error rate p_*ij*_ is computed. S_*ij*_ > 3 and p_*ij*_ < 0.1 are the thresholds used on the BooleanNet statistics to identify Boolean implication relationships. Boolean equivalent relationship between A and B is defined as sparse top-left and bottom-right quadrants (S_01_ > 3, p_01_ < 0.1; S_10_ > 3, p_10_ < 0.1) in the scatterplot between A and B. Boolean equivalent relationships are heavily used in this paper.

### BECC (Boolean Equivalent Correlated Clusters) Analysis

BECC analysis is based on Boolean Equivalent relationships, pair-wise correlation and linear regression analysis (Supplementary Figure S1C). BECC analysis begins with a seed gene. For identification cell cycle genes we used CCNB1 as seed gene (Dabydeen et al., 2019). We used CD14 as a seed gene in this paper. A selected probeset of a seed gene was used as a starting point to identify a list of probesets (ProbeSet A) that are Boolean Equivalent to the selected probeset. Next, this list was expanded (ProbeSet B) by identifying other probesets that are Boolean Equivalent to at least one of the probeset from ProbeSet A. Probeset B were further expanded (ProbeSet C, *L*) by repeating the same steps. All the genes identified in ProbeSet C are used to perform for pair-wise correlation and linear regression analysis. A score was computed for a pair of probesets from *L* by using the correlation *r* and slope of fitted line *s* (if *s* > *1*, *1/s* was used as slope).

$$s\,c\,o\,r\,e=r^{2}+s^{2}$$

The score is a number between 0 and 2 given *r* > 0 and *s* > 0. A matrix of scores *M* was computed for all probesets in *L*. Every row of this matrix was sorted based on the score in ascending order. The whole matrix was then multiplied using a column vector of ranks: *[0 1 2 … len(L)-1]*. In other words, the score for the probeset in row *i gs*_*i*_ was computed as follows:

$$g\,s_{i}=\frac{1}{l\,e\,n\,\left(L\right)}\,\sum_{k=0}^{l\,e\,n\,\left(L\right)-1}k*s\,c\,o\,r\,e_{i\,k}/2$$

where *score*_*ik*_ is the *k*^th^ smallest score for the probeset in row *i*.

StepMiner algorithm was used to compute a threshold to identify the high scoring probesets *gs*_*i*_. The result of the BECC is this list of high scoring probesets.

### Statistical Justification

Empirical distribution of the pair-wise gene scores were computed for each of our dataset by randomly selecting pairs of probesets. Using this distribution, average probeset score E[gs_i_] and standard deviation can be estimated.

$$E\,\left[g\,s_{i}\right]=\frac{1}{l\,e\,n\,\left(L\right)}\,\sum_{k=0}^{l\,e\,n\,\left(L\right)-1}k*\frac{E\,\left[s\,c\,o\,r\,e_{i\,k}\right]}{2}=E\,\left[s\,c\,o\,r\,e\right]*\frac{l\,e\,n\,\left(L\right)-1}{4}$$

$$s\,t\,d\,d\,e\,v\,\left(g\,s_{i}\right)=\sqrt{V\,a\,r\,i\,a\,n\,c\,e\,\left[s\,c\,o\,r\,e\right]*\frac{l\,e\,n\,\left(L\right)-1}{4}}$$

The p-value for the StepMiner identified threshold was computed using a Z-test. All statistical tests were performed using R version 3.2.3 (2015-12-10).

## Results

### BECC Identifies Macrophage Genes in Humans

We previously published a computational tool called Boolean Equivalent Correlated Clusters (BECC) for mining publicly available gene expression datasets (*n* = 25,955 human samples, GSE119087) (Dabydeen et al., 2019). BECC compares the normalized expression of two genes across all datasets by searching for two sparsely populated, diagonally opposite quadrants out of four possible quadrants (high-low and low-high), employing the BooleanNet algorithm (Sahoo et al., 2008). The BECC algorithm only focuses on Boolean Equivalent relationships (Figure 1A and Supplementary Figure S1B) to identify potentially functionally related gene sets (Supplementary Figure S1C).

To use BECC to identify potential macrophage-specific genes, we identified *CD14* as a seed gene as it is expressed in most macrophage populations (Figure 1B) (Griffin et al., 1981; Passlick et al., 1989). However, CD14 is not considered an ideal universal marker of macrophages because of its variable expression patterns among different types of macrophages (Griffin et al., 1981; Passlick et al., 1989). Discovering universal biomarkers for cells like macrophages that reside in many different tissue types and disease states requires large gene expression datasets. For these analyses, we obtained publicly available microarray databases in Human U133 Plus 2.0 (*n* = 25,955, GSE119087) Affymetrix platform from GEO.

The BECC algorithm was first used to identify a set of 9 probesets (ProbeSet A) that were Boolean-Equivalent to the *CD14* gene (201743_at probeset). Then, the same algorithm was used to identify additional probesets that were Boolean-Equivalent to ProbeSet A; pooling the hits in the second step together with those in ProbeSet A resulted in ProbeSet B comprised of 20 probesets. A third step was performed to collect additional candidates and resulted in ProbeSet C with 33 probesets (Figure 1B). BECC computes Boolean Equivalences for three steps because any additional steps have the potential to add significant noise. All probesets in ProbeSet C were then comprehensively analyzed relative to each other to assess the strength of their equivalences. A Boolean-Equivalence score for each probeset within ProbeSet C was computed based on the weighted average of the correlation coefficient and slope in pair-wise analysis with all other probesets, as described in the Methods. This effort resulted in a ranked list of 33 probesets, corresponding to 21 unique genes with similar expression patterns as *CD14*. The entire ranked list of genes can be accessed online using our web-resource. StepMiner, an algorithm which fits a step function to identify abrupt transitions in series data, was used to compute a threshold on the BE score to identify high-confidence macrophage genes. Imposition of the threshold resulted in the identification of 18 significant probesets, representing 13 unique genes (Figure 1C). These 13 genes represent candidates for universal macrophage biomarkers.

We compared CD14 expression patterns with other known markers such as CD16, CD64, CD68, CD71, CCR5 and ITGAM (Supplementary Figures S2A–F). CD14 had better dynamic range compared to these other genes. CD71 was weakly correlated with CD14 suggesting that it may have other tissue specific expression patterns. BECC analyses starting with seed genes CD71 and CCR5 returned no results as none of the genes had Boolean equivalent relationships. CD68 and ITGAM returned too many results, prompting us to increase the threshold (*S* > 50, *p* < 0.1) to generate specificity. Finally, we observed that the results from seed gene CD64 had the most overlap with CD14 (Supplementary Figure S2G). Thus, the BECC results may vary significantly depending which seed gene was used. We prioritized genes with higher dynamic ranges of expression.

### TYROBP and FCER1G Are Two Strong Candidates for Universal Macrophage Biomarkers

**FIGURE 2:**
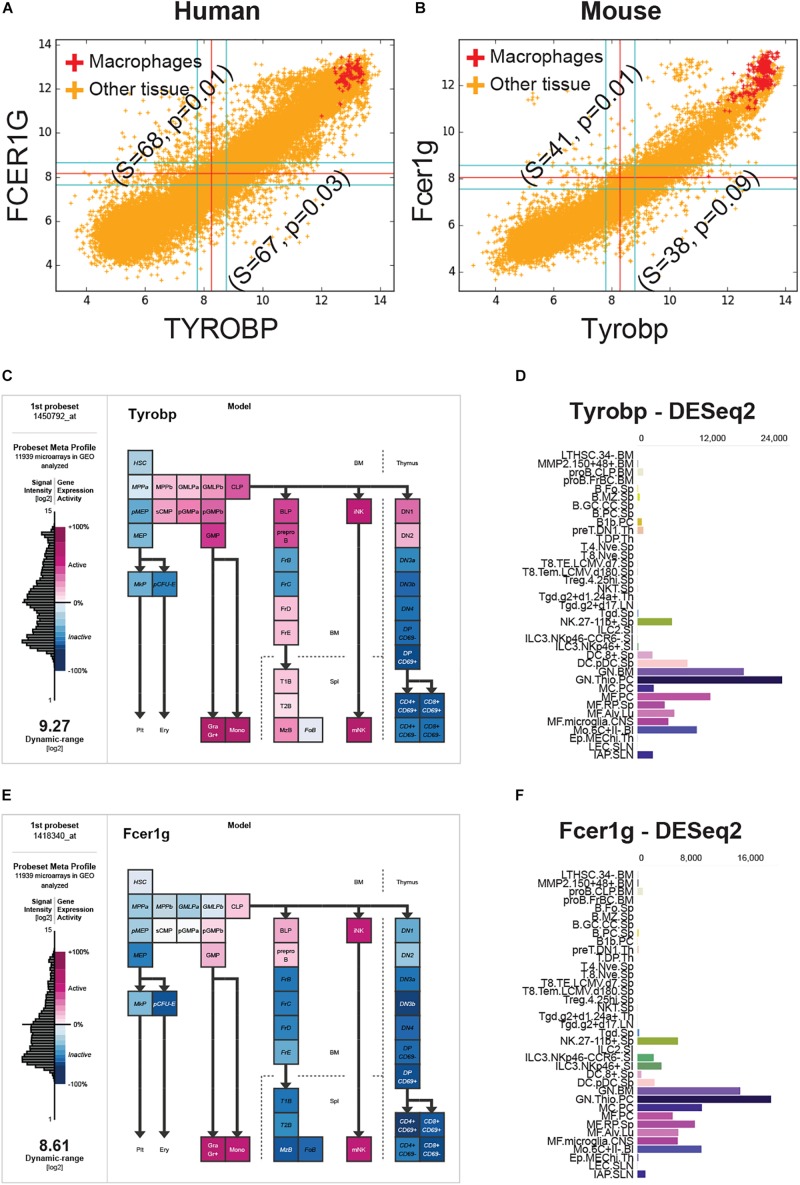
FCER1G and TYROBP expression patterns in human and mouse datasets. **(A)** A scatterplot of TYROBP and FCER1G in human microarray dataset (*n* = 25,955, GSE119087) with macrophage samples (A subset of GSE134312, *n* = 106) are highlighted in red and the rest of them are in orange color. Every point in the scatterplot is a microarray experiment in Human U133 Plus 2.0 Affymetrix platform. **(B)** A scatterplot of Tyrobp and Fcer1g in mouse microarray dataset (*n* = 11,758, GSE119085) in Affymetrix Mouse 430 2.0 platform. Similar to panel A, macrophage samples (GSE135324, *n* = 327) are highlighted in red color and the rest of them are in orange color. **(C)** Expression patterns of Tyrobp in gene expression commons (GEXC). **(D)** Tyrobp gene expression in Immunological Genome Project (ImmGen) ULI RNASeq dataset (GSE127267) obtained using skyline data viewer from ImmGen website. **(E)** Expression patterns of Fcer1g in gene expression commons (GEXC). **(D)** Fcer1g gene expression in ImmGen ULI RNASeq dataset (GSE127267) obtained using skyline data viewer from ImmGen website. **(C,E)** The data is organized in terms of hematopoietic stem cell differentiation hierarchy and heatmap color code is specified in the figure. **(D,F)** Gene skyline from ImmGen shows the different purified hematopoietic cell types that were profiled using RNASeq approach.

FCER1G was the top candidate and TYROBP was the fourth candidate based on the BECC-ranking (Figure 1C). All 13 gene candidates were evaluated on the human and mouse macrophage datasets. FCER1G and TYROBP had the strongest correlation patterns in both human and mouse datasets (Figures 2A,B). We expected that the target biomarkers for macrophages would be highly expressed in pure macrophages sample. Figures 2A,B show scatterplots of TYROBP and FCER1G expression values in both human and mouse datasets, with purified macrophage samples highlighted in red color. We detected high expression of both TYROBP and FCER1G in our carefully annotated macrophage datasets (red color, Figures 2A,B). The orange color samples in Figures 2A,B identified samples from diverse tissue types, including normal, cancer and other diseases. If there are two macrophage-specific genes expressed in all macrophage subtypes in all tissues, their expression pattern would be tightly correlated in bulk tissue datasets because the gene expression values would be proportional to the amount (or number) of macrophages present in each tissue sample. It is evident that their expression pattern is extremely tightly correlated in all bulk gene expression datasets in both human and mouse. This type of expression patterns suggests that TYROBP and FCER1G are expressed in similar contexts in all tissues. We conclude that TYROBP and FCER1G expression patterns are equivalent. Macrophages are present in every tissue, but the number of macrophages varies dramatically between diverse tissue samples. Ideally, a gene that is strongly correlated with the abundance of macrophages in a tissue can be considered as a candidate for a universal macrophage biomarker. However, it is hard to assess the exact number of macrophages in every bulk tissue sample. We observed that TYROBP and FCER1G both are highly expressed in pure macrophage samples (red color, Figure 2) and they are strongly correlated in every tissue samples in human and mouse. Based on this, we hypothesize that TYROBP and FCER1G are universally expressed in all macrophage populations within our datasets. We next tested this hypothesis by validating TYROBP and FCER1G expression in other immune cell types.

We analyzed Tyrobp and Fcer1g expression in GEXC (Figures 2C,E) and ImmGen ULI RNASeq datasets (Figures 2D,F). GEXC (Gene Expression Commons) features 39 distinct highly purified mouse blood cells (GSE34723, *n* = 101) (Seita et al., 2012). ImmGen ULI is an open-source project that features expression profiles of the purified immune cell populations (Painter et al., 2011; Yoshida et al., 2019). We observed that in both datasets, the expression patterns of Tyrobp and Fcer1g were exclusively limited to macrophage-like cells and NK cells. This validates our hypothesis that Tyrobp and Fcer1g are universal candidate biomarkers for mouse macrophages.

### FCER1G and TYROBP Are Highly Expressed in Purified Macrophage Datasets

**FIGURE 3:**
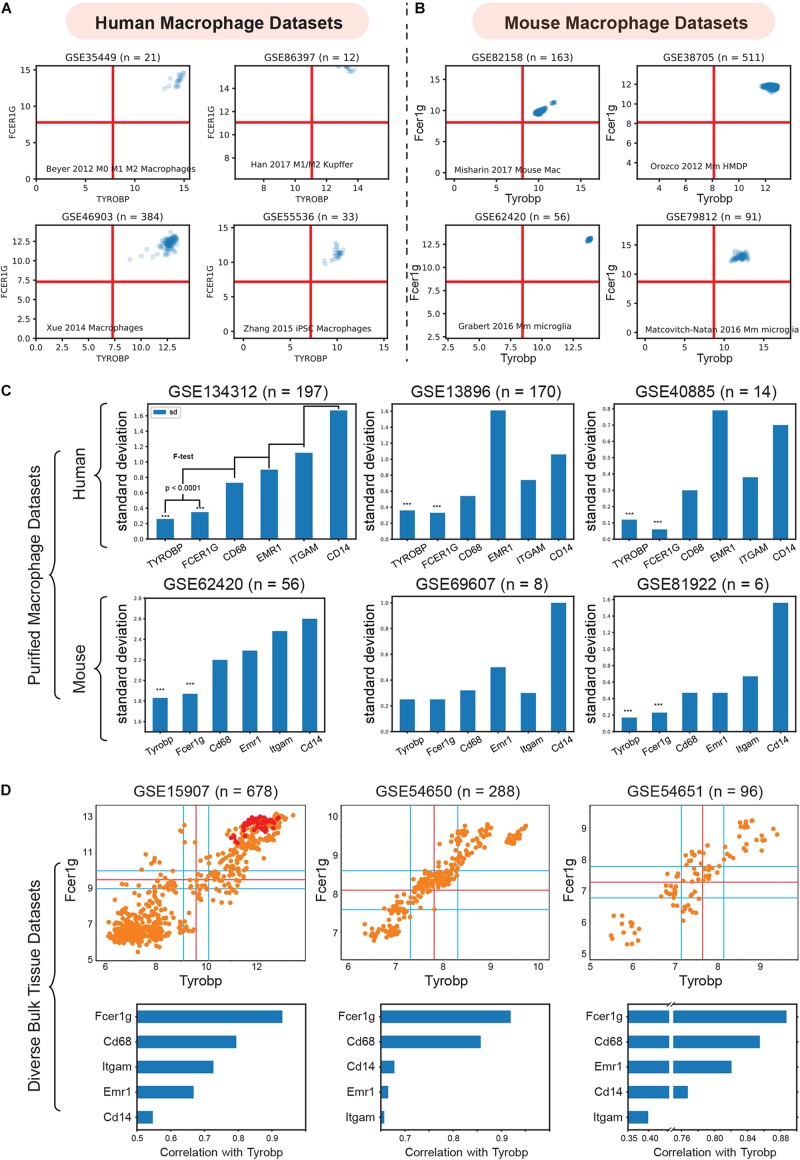
Validation of TYROBP and FCER1G as a universal biomarker of macrophage. **(A)** Expression patterns of TYROBP and FCER1G in four purified human macrophage datasets: (GSE35449, *n* = 21), (GSE85333, *n* = 185), (GSE46903, *n* = 384), (GSE55536, *n* = 33). **(B)** Expression patterns of Tyrobp and Fcer1g in four purified mouse macrophage datasets: (GSE82158, *n* = 163), (GSE38705, *n* = 511), (GSE62420, *n* = 56), and (GSE86397, *n* = 12). **(C)** Standard deviation of TYROBP and FCER1G is compared (*F*-test) to commonly used macrophage biomarker CD68, EMR1, ITGAM, CD14 in purified macrophage datasets in human and mouse, Only pooled macrophage dataset (GSE134312, *n* = 197) was part of training data and the rest are independent validation dataset. **(D)** Pearson’s correlation analysis of Fcer1g, Cd68, Emr1, Itgam, Cd14 with Tyrobp shown as a barplot below the scatterplot between Tyrobp and Fcer1g in three independent bulk tissue datasets. Red colored points represent purified macrophage samples while the orange points represent other cell of tissue types.

To validate TYROBP and FCER1G as universal biomarkers, we interrogated pure macrophage datasets collected from several human and mouse tissues (Figure 3). We combined four purified human macrophage datasets: (GSE35449, *n* = 21) (Beyer et al., 2012), (GSE85333, *n* = 185) (Regan et al., 2018), (GSE46903, *n* = 384) (Xue et al., 2014), (GSE55536, *n* = 33) (Zhang et al., 2015), and four diverse mouse macrophage datasets: (GSE82158, *n* = 163) (Misharin et al., 2017), (GSE38705, *n* = 511) (Orozco et al., 2012), (GSE62420, *n* = 56) (Grabert et al., 2016), and (GSE86397, *n* = 12) (Han et al., 2017).

We then analyzed the diverse human and mouse purified macrophage datasets mentioned above. For each microarray or RNASeq dataset, we computed the range of values observed for different genes and assigned the limits of the *x* and *y*-axis accordingly. The red lines in each plot represent the middle of the range which were used as a threshold to separate high and low values. As shown in Figures 3A,B, all the samples had high-high expression patterns for both TYROBP and FCER1G. This experiment supports our hypothesis and validates TYROBP and FCER1G as candidate biomarkers for human and mouse macrophages.

To test if TYROBP and FCER1G were expressed in human tissue resident macrophages in human, we analyzed nine other datasets (Supplementary Figure S3): (A) tumor associated macrophage (GSE117970, *n* = 13) (Cassetta et al., 2019); (B) lung alveolar macrophages (GSE116560, *n* = 68) (Morrell et al., 2019); (C) lung alveolar macrophages (GSE40885, *n* = 14) (Reynier et al., 2012); (D) cardiac macrophages (GSE119515, *n* = 18) (Dick et al., 2019); (E) vaginal mucosa and skin macrophages (GSE54480, *n* = 70) (Duluc et al., 2014); (F) skin macrophages (GSE74316, *n* = 12) (Carpentier et al., 2016); (G) peritoneal macrophages (GSE79833, *n* = 12) (Irvine et al., 2016); (H) microglia (GSE1432, *n* = 24) (Rock et al., 2005); (I) adipose tissue macrophages (GSE37660, *n* = 2) (Eto et al., 2013). In all cases, we observed have high-high expression patterns for both TYROBP and FCER1G.

We observed differences in expression patterns with respect to skin Langerhans cells (LCs) which are part of the mononuclear phagocyte system and it is reasonable to classify LCs within the macrophage lineage (Deckers et al., 2018). We observed low FCER1G and high TYROBP expression in some human skin LCs (Supplementary Figures S4A,B): (A) human skin Langerhans cells (GSE49475, *n* = 9) (Polak et al., 2014); (B) human skin Langerhans cells (GSE74316, *n* = 13) (Carpentier et al., 2016). However, mouse skin LCs showed high-high expression patterns for both Tyrobp and Fcer1g (GSE74316, *n* = 5) (Carpentier et al., 2016). Dendritic cells (DC) are also mononuclear phagocytes of both lymphoid and myeloid origin. We observed that certain human dermal DCs (CD141+) presented variable expression patterns with respect to FCER1G (GSE74316, *n* = 7) (Carpentier et al., 2016). Despite heterogeneity in FCER1G expression patterns, TYROBP expression patterns remained high in most mononuclear phagocyte cell types.

### FCER1G and TYROBP Performed Better Than ITGAM, CD68, and EMR1

ITGAM (Swirski et al., 2009), CD68(Falini et al., 1993), and EMR1 (F4/80) (Austyn and Gordon, 1981) are currently established universal biomarkers for macrophages. We analyzed gene expression patterns for the above genes and compared them with TYROBP and FCER1G. Our hypothesis was that a universal biomarker should have stable gene expression patterns in pure macrophage samples. We tested this hypothesis using our pooled human macrophage cohorts (GSE134312, *n* = 197) by measuring the standard deviation of gene expression patterns (Figure 3C). TYROBP and FCER1G both had significantly (*p* < 0.0001) lower standard deviation compared to the other established biomarkers. However, since this dataset was part of training data for this analysis, we next used two other independent human datasets GSE13896 (*n* = 170) (Shaykhiev et al., 2009), and GSE40885 (*n* = 14) (Reynier et al., 2012), and three other mouse datasets GSE62420 (*n* = 56) (Grabert et al., 2016), GSE69607 (*n* = 8) (Jablonski et al., 2015), and GSE81922 (*n* = 6) (Jiang et al., 2017). These macrophage datasets had variable expression patterns for the established biomarkers. However, TYROBP and FCER1G had stable, high, and homogeneous expression patterns across diverse macrophage samples. To further demonstrate homogeneity, we performed Pearson’s correlation analysis (Figure 3D) of Tyrobp and Fcer1g in three independent mouse datasets with different tissue and cell types (orange color = tissue sample, red color = purified macrophages sample): GSE15907 (microarray, *n* = 678) (Painter et al., 2011), GSE54650 (microarray, *n* = 288) (Zhang et al., 2014), GSE54651 (RNASeq, *n* = 96) (Zhang et al., 2014). Additionally, a comparison of Fcer1g, Cd68, Emr1, Itgam, and Cd14, revealed that Fcer1g remained the top correlated genes with Tyrobp in these three diverse mouse bulk tissue datasets (Figure 3D).

### FCER1G and TYROBP Are Highly Expressed in Macrophage Single Cell RNASeq Datasets

**FIGURE 4:**
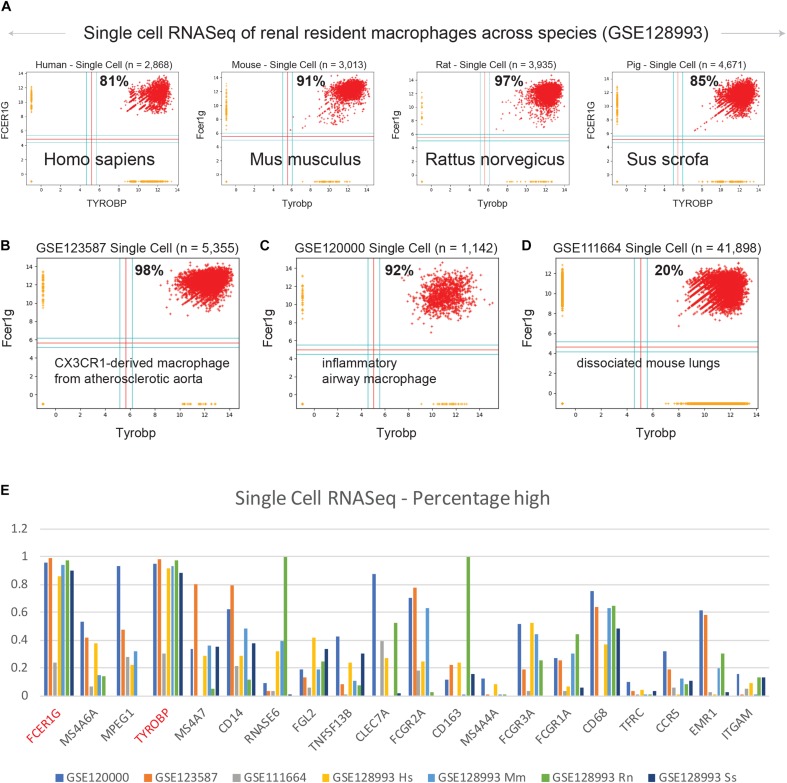
Validation of TYROBP and FCER1G in single cell RNASeq datasets. Scatterplots of expression patterns for TYROBP and FCER1G is shown in several public single cell RNASeq datasets. Red color points denote TYROBP high and FCER1G high samples. Percentage of red points are computed for each scatterplot. Homologous genes are considered for data in mouse, rat and pig. **(A)** renal resident macrophages across species (GSE128993; human *n* = 2,868, mouse *n* = 3,013, rat *n* = 3,935, pig *n* = 4,671), **(B)** mouse CX3CR1-derived macrophage from atherosclerotic aorta (GSE123587; *n* = 5,355), **(C)** mouse inflammatory airway macrophages (GSE120000; *n* = 1,142), and **(D)** mouse dissociated whole lung tissue (GSE111664; *n* = 41,898). **(E)** A bar plot of gene expression values for all 13 genes identified by BECC analysis with seed gene CD14, and the common macrophage genes such as CD16 (FCGR3A), CD64 (FCGR1A), CD68, CD71 (TFRC), CCR5, EMR1, ITGAM, in all the above single cell RNASeq datasets. TYROBP and FCER1G are highlighted in red color.

We examined expression patterns of FCER1G and TYROBP in several publicly available single cell RNASeq datasets (Figure 4): (A) renal resident macrophages across species (GSE128993; human *n* = 2,868, mouse *n* = 3,013, rat *n* = 3,935, pig *n* = 4,671) (Zimmerman et al., 2019), (B) mouse CX3CR1-derived macrophage from atherosclerotic aorta (GSE123587; *n* = 5,355) (Lin et al., 2019), (C) mouse inflammatory airway macrophages (GSE120000; *n* = 1,142) (Mould et al., 2019), and (D) mouse dissociated whole lung tissue (GSE111664; *n* = 41,898) (Aran et al., 2019). We computed the percentage of single cell sample shows high-high expression patterns with respect to both FCER1G and TYROBP. Renal resident macrophages showed 81, 91, 97, and 85% co-expression in human, mouse, rat, and pig respectively (Figure 4A). Mouse CX3CR1-derived macrophages from atherosclerotic aorta and inflammatory airway macrophages showed 98% (Figure 4B) and 92% (Figure 4C) high-high respectively. However, single cell RNASeq data from dissociated mouse whole lungs showed 20% high-high, likely because this sample contained a mixture of cell types including both the epithelial cells and the macrophages. We computed the percentage of samples that demonstrate high expression pattern for all 13 genes identified by BECC analysis with seed gene CD14, and the common macrophage genes such as CD16 (FCGR3A), CD64 (FCGR1A), CD68, CD71 (TFRC), CCR5, EMR1, ITGAM, in the single cell RNASeq datasets (Figure 4E). We observed that TYROBP and FCER1G expression patterns were consistently high in all datasets, and other genes show significant heterogeneity in their expression patterns.

## Discussion

We have developed a computational approach to identify universal genes expressed in diverse macrophage populations. The results are somewhat sensitive to the choice of seed gene. We used CD14 to identify universal macrophages markers. However, choosing alternative seed genes could instead identify markers of macrophage differentiation and polarization, including M1 or M2 cellular phenotypes (Martinez et al., 2006). Seed genes must have good dynamic range and macrophage specificity to perform well. Details of the method, source code and working principles can be found in Supplementary Figure S1. The method filters out asymmetric relationships (Supplementary Figure S2A, CD14 vs. CD16 is an example) and focuses only on the symmetric relationships by using Boolean Implication analysis. The difference between Boolean, correlation and linear regression is that Boolean approach discovers six types of relationships (two symmetric and four asymmetric) whereas correlation and linear regression discovers two types (positive correlation and negative correlation; positive slope and negative slope) of relationships both of which are symmetric. The mathematics used for correlation and linear regression are inherently symmetric. Thus, traditional approaches that are purely based on correlation coefficients or linear regression cannot distinguish symmetric vs. asymmetric relationships (Sahoo et al., 2008). A macrophage differentiation marker will likely define a subset of macrophages and therefore, in the scatterplot between these genes in Y axis and a universal marker in X axis they may follow asymmetric Boolean Implications: X low ⇒ Y low or Y high ⇒ X high.

Using CD14 as seed gene, we discovered TYROBP (TYRO protein tyrosine kinase-binding protein) and FCER1G (Fc fragment of IgE receptor Ig) as ideal candidates for robust, universal macrophage markers. TYROBP is an adapter protein which non-covalently associates with activating receptors found on the surface of a variety of immune cells. TYROBP functions to mediate signaling and cell activation following ligand binding by the receptors (Lanier et al., 1998a, b; Dietrich et al., 2000). Interaction of an allergen with FCER1G triggers cell activation, which induces the release of numerous mediators involved in allergic responses (Blank et al., 2003). Extremely tight correlation was observed between these two genes in all human and mouse macrophage microarray datasets (Figures 2A,B). In the GEXC dataset that contain 39 highly purified cell subsets from mouse blood, Tyrobp and Fcer1g expression were highest in macrophages and NK cells (Figures 2C,E). B cell and T cell progenitors also show slightly higher expression patterns for Tyrobp and Fcer1g compared to other cell subset such as hematopoietic stem cell (HSC), megakaryocyte (MkP) and erythrocyte (pre-CFU-E) progenitors. Immgen skyline data viewer restricted Tyrobp and Fcer1g expression patterns to granulocytes, microglia and macrophages (Figures 2D,F). Immgen data showed low expression in natural killer (NK) and dendritic cells (DCs). Both PBMC-derived and tissue resident macrophages showed high expression for TYROBP and FCER1G in diverse settings including single-cell data, adding significant strength to our hypothesis (Figures 3, 4). TYROBP and FCER1G emerged as superior in direct head-to-head comparison with all 13 genes identified by BECC using CD14 as seed gene, and common macrophage markers such as CD16, CD64, CD68, CD71, CCR5, EMR1 and ITGAM (Figure 4D). One exception was found in human skin Langerhans cells and dermal dendritic cells which showed FCER1G low and TYROBP high (Supplementary Figure S4). These data suggested that TYROBP is superior to FCER1G in identifying all mononuclear phagocytes in human samples irrespective of their lymphoid or myeloid origin. Further validation is needed to establish TYROBP and FCER1G as universal markers of macrophages. Literature review showed a computational approach named correlation-based feature subset (CFS) identified TYROBP as part of the hub genes in kidney cancer samples using protein-protein interaction network (Wang et al., 2019). Another study reported that microglia in IDH-mutants are mainly pro-inflammatory, while anti-inflammatory macrophages that upregulate genes such as FCER1G and TYROBP predominate in IDH-wild type GBM (Poon et al., 2019). Tyrobp and Fcer1g was found to be differentially expressed in Alzheimer’s disease (AD) mouse models that demonstrated strong correlation between cortical Aβ amyloidosis and the neuroinflammatory response (Castillo et al., 2017). FCER1G was part of a hub gene in a meta-analysis of lung cancer samples (Guo et al., 2019).

Normalization is key to perform a reliable high-throughput data analysis. To perform large scale gene expression analysis, all samples from a dataset must be in the same measurement platform. Microarray and RNASeq technologies allow the monitoring of expression levels for thousands of genes simultaneously. However, in these experiments, many undesirable systematic variations are observed even in replicated experiments. Normalization is the process of removing some sources of variation which affect the measured gene expression levels. It is easier to normalize microarray data in one platform. It is much harder to normalize data across platforms due to platform-related technical bias. We have pooled all publicly available Affymetrix datasets in U133A, U133A_2 and U133 Plus 2.0 platform for human samples, and in Affymetrix Mouse Genome 430 2.0 Array for mouse samples. We normalized all Affymetrix microarrays using RMA (Robust Multiarray Average) in their respective platforms separately (Irizarry et al., 2003a, b). However, Affymetrix datasets in U133A, U133A_2 and U133 Plus 2.0 were pooled into one dataset by using a modified CDF file that contains shared probesets from these three different platforms.

Macrophage dysfunction can lead to many human diseases and pathologies, including impaired wound healing, fibrosis (Murray and Wynn, 2011), chronic inflammatory diseases (Kamada et al., 2008; Hansson and Hermansson, 2011; Murray and Wynn, 2011), diabetic complications (Huang et al., 2010; Eguchi et al., 2012), and cancer (Qian and Pollard, 2010). They play central roles during development (Pollard, 2009), homeostatic tissue processes (Wynn et al., 2013), tissue repair (Wynn et al., 2013), and immunity (Phan et al., 2017). Macrophages play a vital role in chronic inflammatory diseases such as atherosclerosis (Hansson and Hermansson, 2011) and chronic kidney disease (Henaut et al., 2019). Due to their large involvement in the pathogenesis of several types of human diseases, macrophages are relevant therapeutic targets (Advani et al., 2018). Macrophage biology, mechanisms of action, and activation phenotypes have been studied extensively in recent years. Macrophages have a strong tendency to adapt to the microenvironment and to rapidly change in response to environmental stimuli. Thus, it is difficult to design a unique therapeutic strategy based on macrophage modulation that is easily applicable to different kinds of human pathologies. However, our approach appears to identify universal biomarkers that restrict macrophages to a homogeneous state. Our experiments suggest that the variable expression patterns demonstrated by the established macrophage biomarkers is seen within macrophages across different tissues. However, in sharp contrast, TYROBP and FCER1G maintain homogeneity of expression patterns within macrophages across different tissues. These candidates would be golden targets of several human diseases as the macrophages would have hard time adapt to any intervention that targets their fundamental properties. The proposed method can be applied in other biological context following the success of macrophage targeting.

## Data Availability Statement

All the data generated in the described analyses are submitted to GEO: GSE119085 (mouse), GSE119087 (human), GSE119128 (collections), GSE134312 (human macrophages), and GSE135324 (mouse macrophages).

## Data Access

GSE119085 – Mouse Boolean Implication Network.

GSE119087 – Human Boolean Implication Network.

GSE119128 – An unbiased Boolean analysis of public gene expression data for cell cycle gene classification.

GSE134312 – Pooled macrophage datasets from GEO.

GSE135324 – Pooled mouse macrophage datasets from GEO.

## Author Contributions

DS contributed to the conceptualization, the data curation, the computation, the formal analysis, the investigation, the methodology, the project administration, the validation, the visualization, the writing of the original draft, the review and editing of the manuscript, the funding acquisition, the resources, and the supervision. LP contributed to the review and editing of the manuscript, the funding acquisition, and the resources. PG contributed to the data curation, the analysis, the validation, the review and editing of the manuscript, the funding acquisition, and the resources. SD contributed to the data curation, the validation, the review and editing of the manuscript, the funding acquisition, and the resources. ST contributed to the data curation, validation, and writing. DD contributed to the coordination, the data curation, the investigation, analysis, the validation, and the writing of the manuscript.

## Conflict of Interest

The authors declare that the research was conducted in the absence of any commercial or financial relationships that could be construed as a potential conflict of interest.

## Abbreviations

BECC: Boolean Equivalent Correlated Clusters
GEO: Gene Expression Omnibus
ImmGen: Immunological Genome Project
NCI: National Cancer Institute
NIH: National Institute of Health.
