# Supplementary material for: Computational Approach to Identifying Universal Macrophage Biomarkers
Source: Front Physiol. 2020 Apr 8;11:275. doi: 10.3389/fphys.2020.00275 (PMC7156600; doi:10.3389/fphys.2020.00275)
Supplement: Supplementary file 1 [file Data_Sheet_1.PDF]

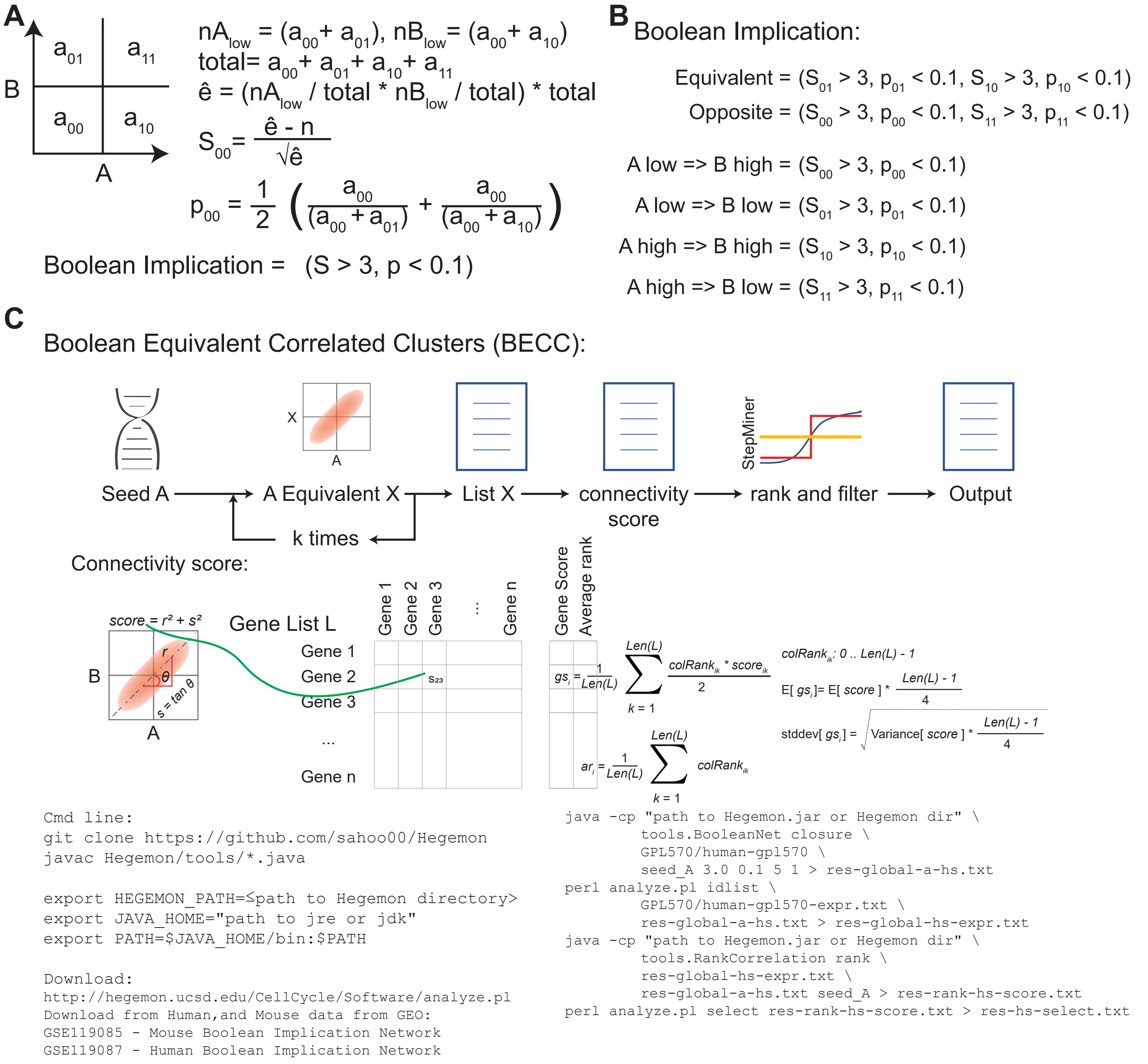

Figure S1: Computational approaches for Boolean analysis: (A) BooleanNet statistic. Evaluating Boolean implication relationship between gene A and B. a_{ij} is the number of samples in the respective quadrants. nA/B_{low} is number of samples where A/B is low. S_{00} = BooleanNet statistic and and p_{00} = error rate to test sparsity for the bottom left quadrant. $S > 3$ and $p < 0.1$ is used to test whether each quadrant is sparse. (B) Deriving Boolean implication relationships using BooleanNet statistic. (C) Workflow and detailed steps of the BECC (Boolean Equivalent Correlated Clusters) tool. A seed gene A is used to extract a list of genes L that are connected by Boolean equivalent relationship directly or indirectly depending on the number k times the loop is considered. A connectivity score is computed for each gene in list L by using the matrix of scores between all pairs that determines how tightly a gene is related to the cluster of genes in L. A gene score is computed as weighted average of the column ranks for each gene. Average gene rank is also computed for each gene which is used to rank genes. StepMiner is used to put a threshold on the gene score to filter highly ranked genes. Output is the candidate gene list computed by BECC.
