# Supplementary material for: Computational Approach to Identifying Universal Macrophage Biomarkers
Source: Front Physiol. 2020 Apr 8;11:275. doi: 10.3389/fphys.2020.00275 (PMC7156600; doi:10.3389/fphys.2020.00275)
Supplement: Supplementary file 2 [file Data_Sheet_2.PDF]

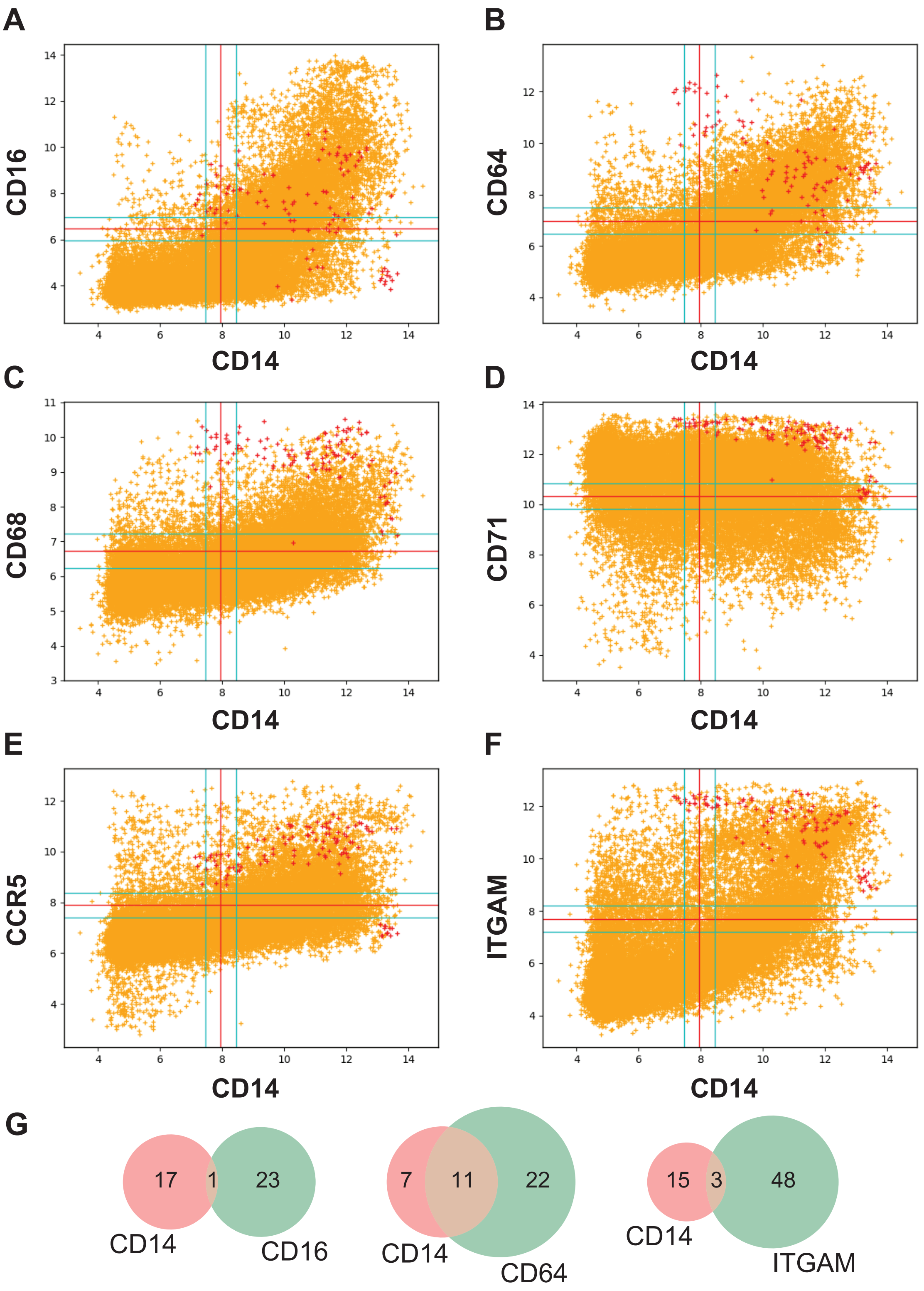

Figure S2: Relationship between CD14 and other known macrophage markers: Scatter plots of gene expression data between CD14 (201743_at) and other known macrophage markers in global human dataset (GSE119087). Red points corresponds to purified macrophage samples (GSE134312). Orange points correspond to other human samples. (A) CD14 vs CD16 (204006_s_at). (B) CD14 vs CD64 (216950_s_at). (C) CD14 vs CD68 (203507_at). (D) CD14 vs CD71 (208691_at). (E) CD14 vs CCR5 (206991_s_at). (F) CD14 vs ITGAM (205786_s_at). (G) Overlap between the BECC analyses based on different seed genes. BECC analyses on CD71 and CCR5 returned no results. BECC on ITGAM and CD68 returned too many results, therefore we increased BooleanNet statistic to $S > 50$, $p < 0.1$ for these two genes.
