# Supplementary material for: Computational Approach to Identifying Universal Macrophage Biomarkers
Source: Front Physiol. 2020 Apr 8;11:275. doi: 10.3389/fphys.2020.00275 (PMC7156600; doi:10.3389/fphys.2020.00275)
Supplement: Supplementary file 3 [file Data_Sheet_3.PDF]

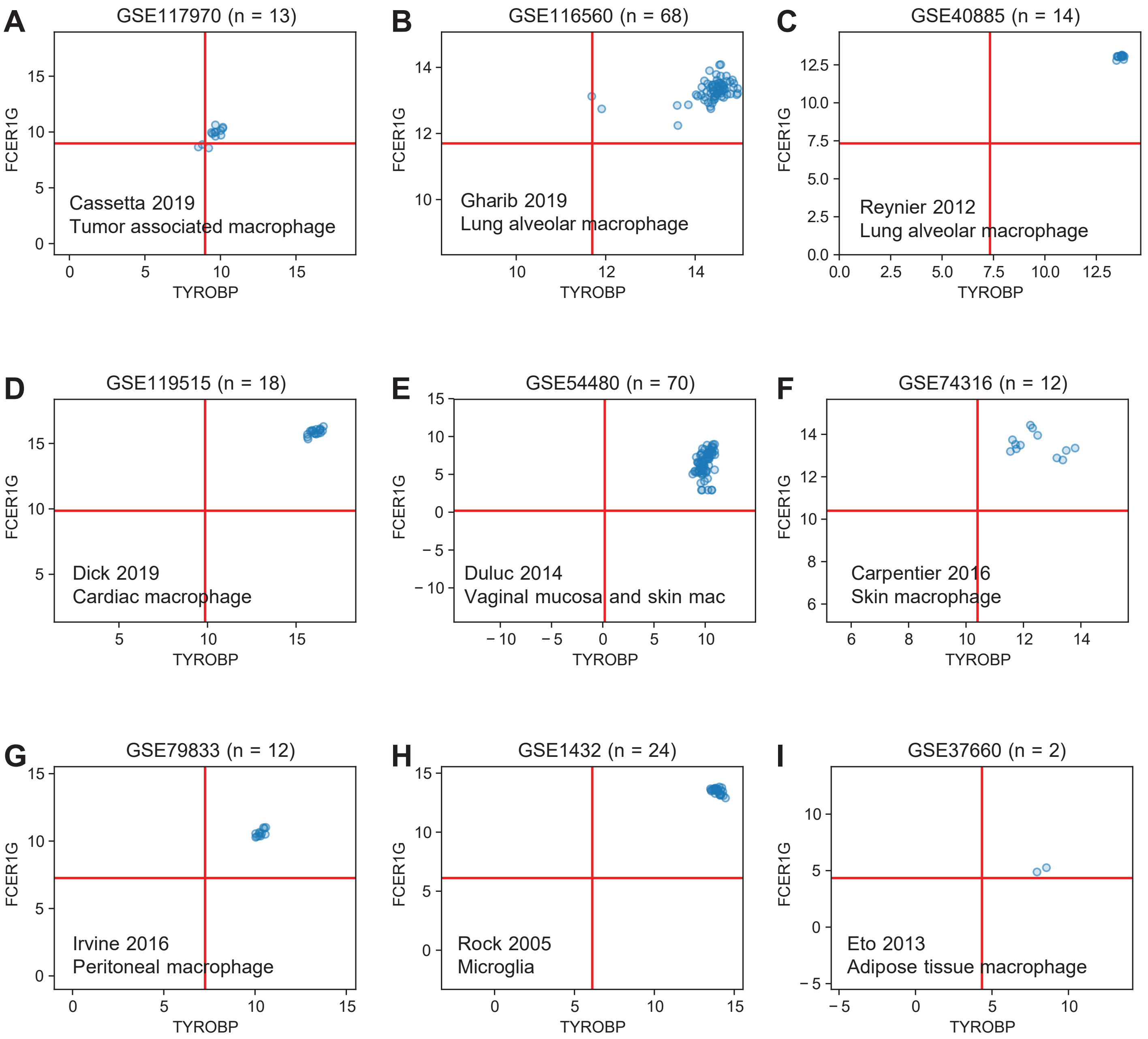

Figure S3: TYROBP and FCER1G expression in human tissue resident macrophages. The limits of the axes were set to the minimum and maximum expression values in each dataset. The red lines denotes the mid point between the minimum and maximum values. Scatter plots of TYROBP and FCER1G in human tissue resident macrophages in nine different context: (A) tumor associated macrophage (GSE117970, n = 13); (B) lung alveolar macrophages (GSE116560, n = 68); (C) lung alveolar macrophages (GSE40885, n = 14); (D) cardiac macrophages (GSE119515, n = 18); (E) vaginal mucosa and skin macrophages (GSE54480, n = 70); (F) skin macrophages (GSE74316, n = 12); (G) peritoneal macrophages (GSE79833, n = 12); (H) microglia (GSE1432, n = 24); (I) adipose tissue macrophages (GSE37660, n = 2).
