# Supplementary material for: Computational Approach to Identifying Universal Macrophage Biomarkers
Source: Front Physiol. 2020 Apr 8;11:275. doi: 10.3389/fphys.2020.00275 (PMC7156600; doi:10.3389/fphys.2020.00275)
Supplement: Supplementary file 4 [file Data_Sheet_4.PDF]

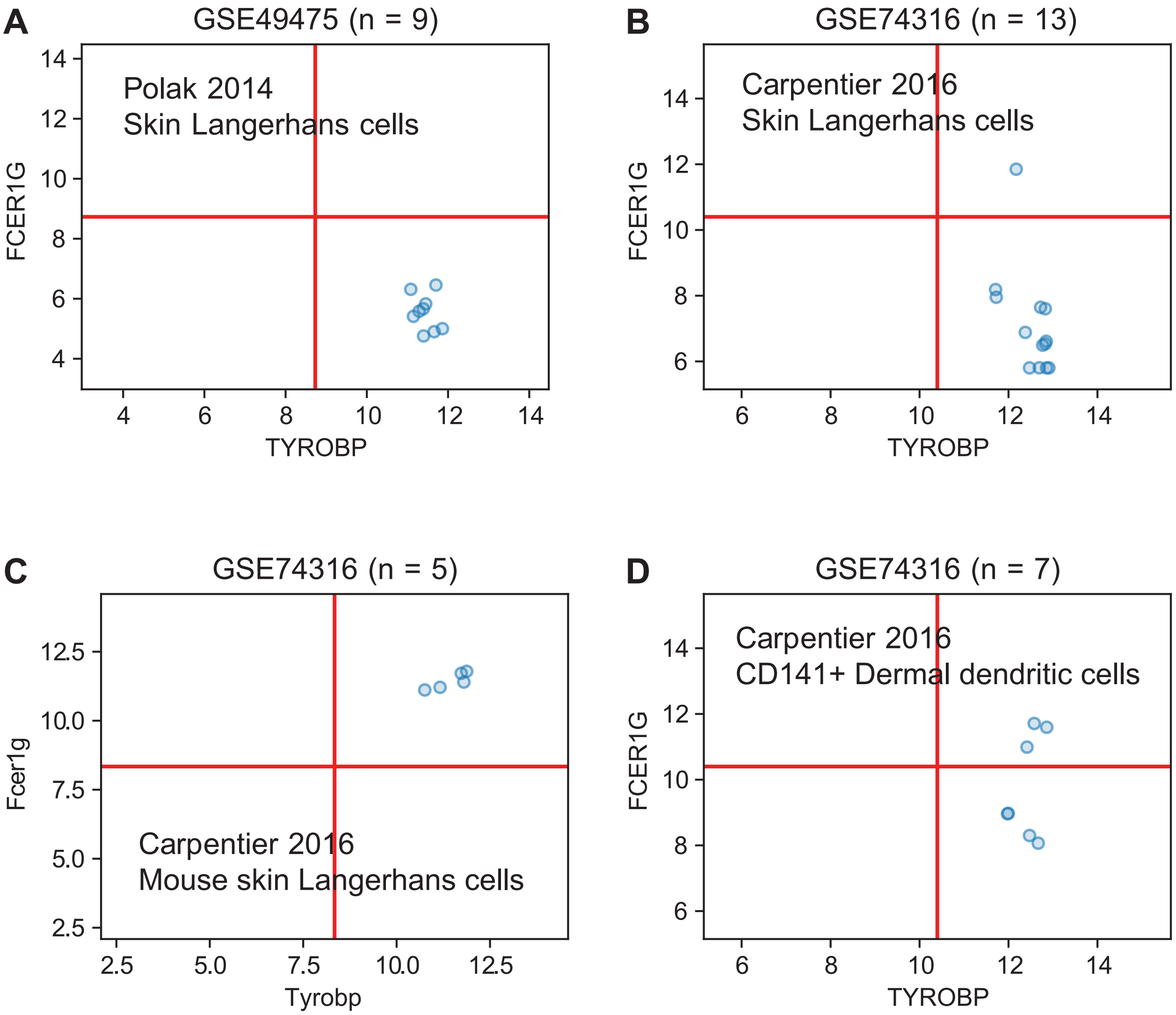

Figure S4: TYROBP and FCER1G expression in skin LCs and DCs. The limits of the axes were set to the minimum and maximum expression values in each dataset. The red lines denotes the mid point between the minimum and maximum values. Scatter plots of TYROBP and FCER1G in skin Langerhans cells and dendritic cells: (A) human skin Langerhans cells (GSE49475, n = 9); (B) human skin Langerhans cells (GSE74316, n = 13); (C) mouse skin Langerhans cells (GSE74316, n = 5); (D) human CD141+ dermal dendritic cells (GSE74316, n = 7).
